# Supplementary material for: Genetic and Genomic Analysis Identifies bcltf1 as the Transcription Factor Coding Gene Mutated in Field Isolate Bc116, Deficient in Light Responses, Differentiation and Pathogenicity in Botrytis cinerea
Source: Int J Mol Sci. 2025 Apr 8;26(8):3481. doi: 10.3390/ijms26083481 (PMC12027217; doi:10.3390/ijms26083481)
Supplement: Supplementary file 1 [file ijms-26-03481-s001.zip › Table S2. Oligonucleotides used in this work.pdf]

Table S2. Oligonucleotides used in this work. The underlined sequences show the *attB* sites.

Name	Sequence 5' -> 3'
bcltf1-c2F	<u>GGGGACAAGTTTGTACAAAAAAGCAGGCT</u>TACTCACAATACAACACAGCACAGC
bcltf1-c2R	<u>GGGGACCACTTTGTACAAGAAAGCTGGGT</u>ACCGCGATTCAACTTAGATGTTGG
PoliC- F5′	CTGCAGCTGTGGAGCCGC
HphF	GCGCTTCTGCGGGCGATTTG
HphR	CGGGTTCGGCCCATTCGGAC
