# Supplementary figures and images for: Genetic and Genomic Analysis Identifies bcltf1 as the Transcription Factor Coding Gene Mutated in Field Isolate Bc116, Deficient in Light Responses, Differentiation and Pathogenicity in Botrytis cinerea
Source: Int J Mol Sci. 2025 Apr 8;26(8):3481. doi: 10.3390/ijms26083481 (PMC12027217; doi:10.3390/ijms26083481)

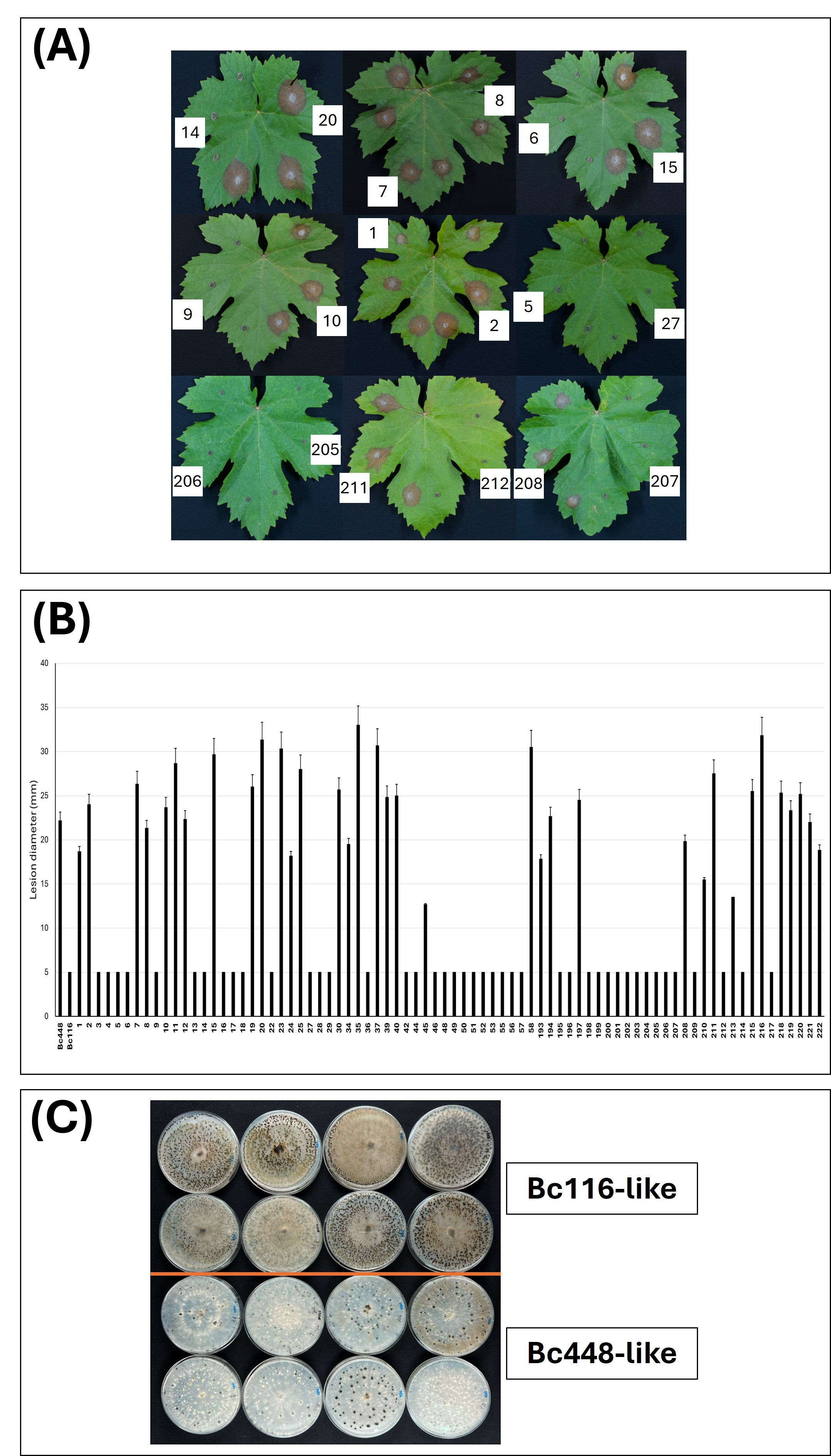

Supplement: Supplementary file 1 [file ijms-26-03481-s001.zip › Fig. S1.tiff]
